# Open label comparative trial of mono versus dual antibiotic therapy for Typhoid Fever in adults

**DOI:** 10.1371/journal.pntd.0006380

**Published:** 2018-04-23

**Authors:** Niv Zmora, Sudeep Shrestha, Ami Neuberger, Yael Paran, Rajendra Tamrakar, Ashish Shrestha, Surendra K. Madhup, T. R. S. Bedi, Rajendra Koju, Eli Schwartz

**Affiliations:** 1 Tel Aviv Sourasky Medical Center, Tel Aviv, Israel; 2 Sackler Faculty of Medicine, Tel Aviv University, Tel Aviv, Israel; 3 Dhulikhel Hospital, Kathmandu University Hospital, Dhulikhel, Nepal; 4 Travel Medicine & Tropical Diseases and Internal Medicine B, Rambam Medical Center, Haifa, Israel; 5 Bruce Rappaport Faculty of Medicine, Technion, Haifa, Israel; 6 The Center for Geographic Medicine and Tropical Diseases, the Chaim Sheba Medical Center, Tel Hashomer, Israel; Oxford University Clinical Research Unit Vietnam, VIET NAM

## Abstract

**Background:**

Emerging resistance to antibiotics renders therapy of Typhoid Fever (TF) increasingly challenging. The current single-drug regimens exhibit prolonged fever clearance time (FCT), imposing a great burden on both patients and health systems, and potentially contributing to the development of antibiotic resistance and the chronic carriage of the pathogens. The aim of our study was to assess the efficacy of combining third-generation cephalosporin therapy with azithromycin on the outcomes of TF in patients living in an endemic region.

**Methods:**

An open-label, comparative trial was conducted at Dhulikhel Hospital, Nepal, between October 2012 and October 2014. Only culture-confirmed TF cases were eligible. Patients were alternately allocated to one of four study arms: hospitalized patients received either intravenous ceftriaxone or a combination of ceftriaxone and oral azithromycin, while outpatients received either oral azithromycin or a combination of oral azithromycin and cefexime. The primary outcome evaluated was FCT and the secondary outcomes included duration of bacteremia.

**Results:**

105 blood culture-confirmed patients, of whom 51 were treated as outpatients, were eligible for the study. Of the 88 patients who met the inclusion criteria for FCT analysis 41 patients received a single-agent regimen, while 47 patients received a combined regimen. Results showed that FCT was significantly shorter for the latter (95 versus 88 hours, respectively, p = 0·004), and this effect was exhibited in both the hospitalized and the outpatient sub-groups. Repeat blood cultures, drawn on day 3, were positive for 8/47 (17%) patients after monotherapy, versus 2/51 (4%) after combination therapy (p = 0·045). No severe complications or fatalities occurred in any of the groups.

**Conclusions:**

Combined therapy of third-generation cephalosporins and azithromycin for TF may surpass monotherapy in terms of FCT and time to elimination of bacteremia.

**Trial registration:**

Trial registration number: NCT02224040.

## Author summary

Typhoid fever (TF) is a serious disease and the most common etiology of bloodstream infections in febrile patients in the Indian subcontinent. Before the advent of antibiotics its mortality rate reached up to 40%, and dropped dramatically upon their introduction. However, over the last decades multidrug-resistant strains have emerged, further posing a challenge to the treatment of TF. Here, we propose a novel treatment approach, combining azithromycin and a third-generation cephalosporin, two antibiotic agents, which act synergistically on the two niches occupied by the bacteria, the intra- and the extra-cellular compartments respectively. In our study of a rural Nepalese population with culture-confirmed TF, we have shown that dual therapy was superior to monotherapy in terms of time to defervescence and bacteremia elimination, both in outpatient and inpatient settings. We hence advocate the combination of these two antibiotics as a more effective therapeutic strategy than the current standard of care, and suggest that such approach may shorten patients’ hospital stay, and reduce both pathogen carriage rates and the development of antibiotic resistance.

## Introduction

Typhoid (Enteric) Fever (TF) is a human-restricted disease caused by the pathogens *Salmonella enterica* serovar Typhi (*S*. Typhi) and Paratyphi (*S*. Paratyphi), which is associated with significant morbidity and mortality when untreated. The disease is transmitted through a fecal-oral route via contaminated food and water, and is therefore a marker of poverty and lack of proper infrastructure. Although rare in developed countries, it is highly endemic in developing countries, mainly in the Indian subcontinent, where it peaks during the monsoon months (June to August) and imposes a great burden on health economies. According to the WHO, the estimated global incidence of TF is about 21 million cases, resulting in more than 200,000 deaths annually [www.who.int/immunization/diseases/typhoid/en/].

Nepal suffers greatly from a lack of sanitary conditions, an abundance of natural disasters and political instability, perpetuating its notorious reputation as a global TF hub. Numerous studies have demonstrated that TF accounts for a large proportion of febrile illnesses in the vicinity of the capital Kathmandu, with a range of one-third to three-quarters of culture-proven etiologies[1–3].

Over the years a variety of antibiotic agents were used to treat TF[4]. Initially, chloramphenicol was sufficient to eradicate the bacteria; however due to the emergence of plasmid-mediated resistance in the 1950s treatment was switched to ampicillin and co-trimoxazole. In the late 1980s these agents too were abandoned following plasmid-mediated resistance, which made the pathogen multidrug resistant (MDR). In the 1990s fluoroquinolones were introduced as effective alternative agents and became the treatment of choice for TF. However, indiscriminate use of antibiotics led to selective pressure for chromosomal mutations in the bacteria, inducing resistance to nalidixic acid (NA) and decreasing their susceptibility to fluoroquinolones, thus necessitating prolonged treatment courses and increased doses[5]. Though treatment with gatifloxacin, a fourth-generation quinolone, was used for a short period of time in the early 2000s, a recent study from Nepal underlined high-level resistance to this regimen[6,7]. Correspondingly, a recent comprehensive report on TF cases in the United States, most of which were contracted in South Asia, has delineated a high and steadily increasing rate of NA resistance[8]. More recent guidelines[9]^,^[10] have recommended the use of azithromycin or third-generation cephalosporins, albeit to a lesser degree of efficacy in terms of fever clearance time (FCT), exhibiting failure rates exceeding 20% of treated cases in some shot-term regimens[11]^,^[12]. These ongoing trends in antibiotic resistance profiles have grave epidemiological consequences, as indolent clinical responses to treatment, prolonged time to defervescence and increased fecal carriage rates translate into greater transmission potential and impede source control[11].

Dual antibiotic treatment for TF is a novel paradigm to improve therapeutic outcomes and reduce the emergence of antibiotic resistance. A recent study performed *in vitro* has shown that the combination of cefotaxime and ciprofloxacin against NA-resistant strains of *S*. Typhi and *S*. Paratyphi exhibited a synergistic effect[13]^,^[14]; and another recent case report has proposed a combination therapy of meropenem and fosfomycin for highly drug-resistant *S*. Typhi in a traveler returning from India[15]. Conversely, a combination of ofloxacin and azithromycin has been assessed in Vietnamese children and has not proven superior to either agent administered separately, presumably because the isolated bacterial strains were highly resistant to NA[16]. A study conducted among Israeli travelers returning from Nepal after the outbreak of *S*. Paratyphi A in 2009 showed that dual therapy with ceftriaxone and azithromycin was vastly superior to monotherapy with intravenous ceftriaxone alone, especially in terms of time to defervescnece, as FCT was shortened by more than 50% (three versus six days) in patients who received dual therapy compared to monotherapy[17]. However, it should be noted that in the Israeli study sample size was small, the study population consisted of travelers, who had not been exposed to TF in the past, and the infecting *Salmonella* strain was identical in all cases. Therefore, dual antibiotic therapy warrants further research before these findings can be applied to populations of endemic areas[18].

In light of such sparse experimental evidence, we set out to compare the efficacy of dual antibiotic regimens consisting of a third-generation cephalosporin and azithromycin to treatment with each of these agents alone for uncomplicated TF in typhoid-endemic areas, and hypothesized that combination therapy would outperform monotherapy in terms of FCT and bacteremia elimination rate.

## Methods

### Study design and participants

A multiarm, parallel, open-label, comparative trial was conducted on adult patients, 18 years of age or older, who attended Dhulikhel Hospital, Nepal, between October 2012 and October 2014. Dhulikhel hospital is an independent non-governmental, non-profit, institution, 30 kilometers northeast of Kathmandu. It accommodates a mostly rural population from the surrounding villages and has 475 beds.

Only subjects with blood cultures positive for *S*. Typhi or *S*. Paratyphi were eligible. Exclusion criteria included known allergies to cephalosporins or macrolides, inability to swallow oral medications, antibiotic treatment within four days prior to admission, significant underlying illnesses and pregnancy or lactation at the time of enrollment.

### Ethics statement

The study protocol was reviewed and approved by Kathmandu University School of Medical Sciences Institutional Review Committee (KUSMS/IRC) and by the Nepalese Health Research Council (study ID number 64/12).

### Allocation process and assignment to treatment arms

Febrile adult patients attending the emergency room (ER) or outpatient department (OPD), who were clinically suspected of having TF by Dhulikhel Hospital’s physicians and who complied with the study inclusion and exclusion criteria, were given a detailed explanation regarding the study and asked to sign written informed consent forms. Case definition for suspected TF included undifferentiated fever lasting more than 48–72 hours prior to antibiotic treatment. Venous blood cultures were drawn at the time of enrollment from every patient meeting these criteria.

Patients were initially assigned into either an inpatient or an outpatient setting, according to their general appearance and the severity of their symptoms, as perceived by the examining physicians, and based on their personal preference and financial ability. They were then allocated to two treatment arms, monotherapy versus dual therapy, according to the order of arrival in an equal allocation ratio. Treatment arms for inpatients were an intravenous 2-gram dose of ceftriaxone once daily (OD) versus a combination of an intravenous 2-gram dose of ceftriaxone OD plus oral azithromycin 500 mg OD; treatment arms for outpatients were oral azithromycin 500 mg OD versus a combination of oral azithromycin 500 mg OD plus oral cefixime 400 mg OD. Neither the patients nor the medical personnel were blinded to the assignment into groups.

Once blood culture results were available, patients with proven *S*. Typhi or *S*. Paratyphi bacteremia were included in the study and were asked to fill out a demographic questionnaire. Patients with negative cultures were excluded from the study and received standard care.

Antibiotic treatment was administered for 7 days or 72 hours following defervescence (whichever was longer). Inpatients were normally discharged from hospital 48 hours following defervescence or 24 hours following defervescence upon request. In either case they were asked to complete a 7-day antibiotic course. When discharged, the patients were given oral cefixime for the remainder of the treatment instead of intravenous ceftriaxone. In case of persistent fever, treatment was extended as deemed necessary by the attending physician.

### Data collection

Demographic data, including age, gender, occupation and place of residence, along with presenting symptoms and medical history were collected through a questionnaire. Physical examination was performed by a qualified physician.

Inpatients had their vital signs taken and underwent physical examination twice daily by the Dhulikhel Hospital Internal Medicine ward staff. The vital signs of outpatients were recorded at 12-hour intervals by trained community medical auxiliaries during house calls, or the patients alternatively attended nearby clinics and pharmacies.

Blood tests and cultures were initially collected at enrollment and then on day three. Patients with persistent bacteremia on day three had a third blood culture drawn on day five as well. Blood was cultured in the hospital’s microbiological laboratory by use of BACTEC radiometric blood cultures. Testing of isolates for susceptibility to various antibiotics was carried out by the disc diffusion method, and in case of resistance to the assigned regimen, treatment was switched accordingly and the patient was excluded from the study.

One month following discharge patients were asked to return for vital signs recording, physical examination and stool cultures to assess fecal carriage of the pathogen and check for relapse.

### Outcomes

The primary endpoint of our trial was FCT, defined as the time from the first dose of antibiotics treatment until oral temperature dropped ≤ 37·5 degrees Celsius for at least 48 hours. The use of paracetamol was restricted to pain relief and not fever alleviation, and FCT was determined after confirmation that the patient had not taken paracetamol 12 hours prior to vital signs measurement. Secondary endpoints were bacteremia clearance time, assessed by the proportion of patients who cleared bacteremia by three and five days after start of treatment; treatment failure, defined as the need to switch antibiotic treatment according to the physician’s decision; development of TF-related complications; late relapse; fecal carriage and adverse drug reactions.

### Data analysis

Power analysis based on data retrieved from an Israeli study[17] indicated a minimum sample size of 88 patients (divided into 4 treatment groups of 22 each), assuming a 36-hour difference in time to defervescence between treatment groups with standard deviation (SD) of 40 hours and given alpha error probability of 0·05 and power of 0·90, based on previous published literature[1–3].

Data were analyzed with Prism 7·0 software (GraphPad Software Inc., La Jolla, CA, USA).

Differences between groups in terms of FCT were evaluated with a log-rank (Mantel-Cox) test. Multiple groups were compared by one-way ANOVA with a Tukey post-hoc test. Differences between groups in terms of clearance of bacteremia were assessed using Fischer’s exact test.

Data are presented as means ± SD, and p < 0·05 was considered statistically significant.

## Results

**Fig 1:**
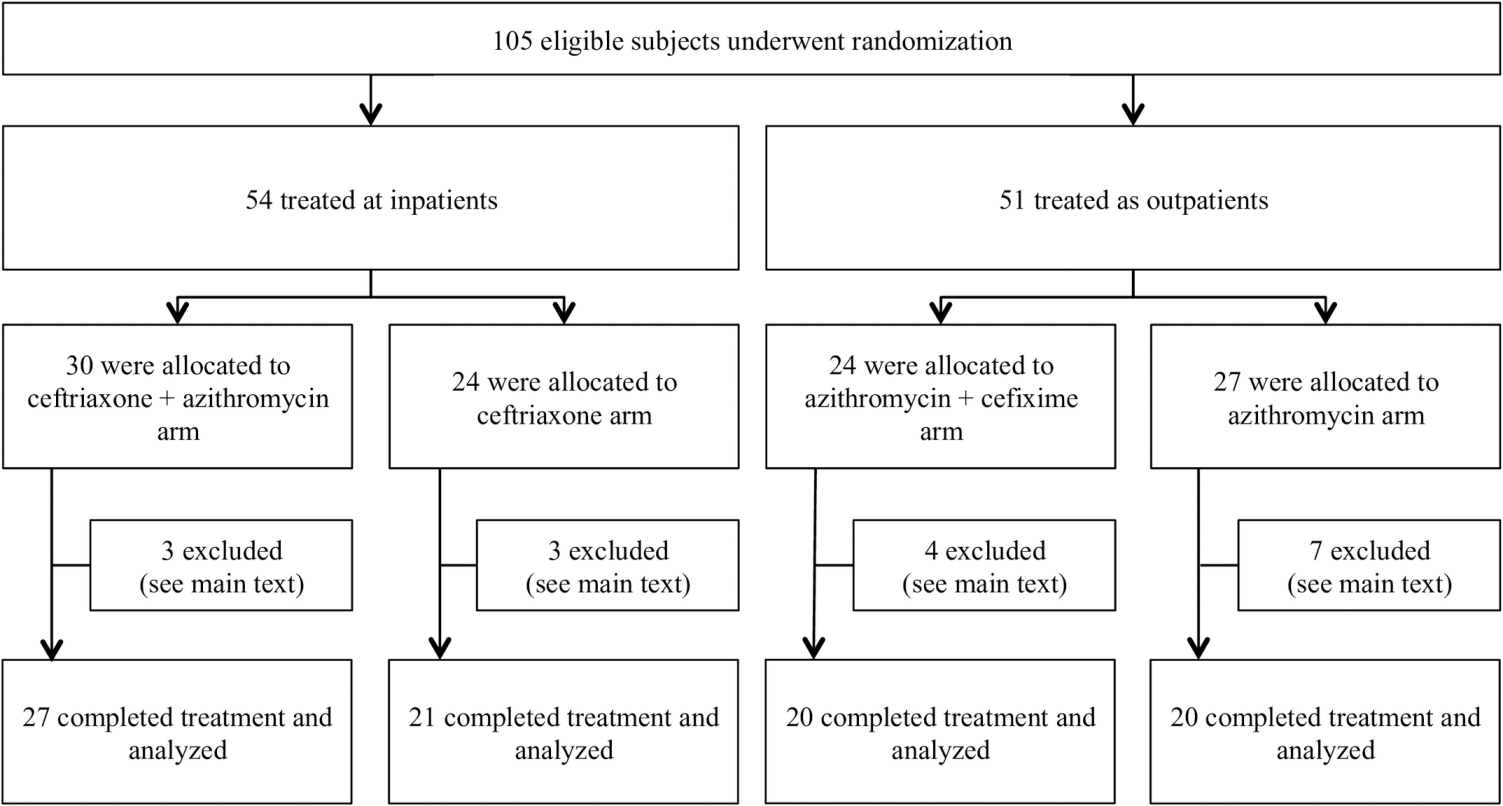
Study flow diagram of patients with blood cultures positive for *S*. Typhi and *S*. Paratyphi.

Between October 2012 and October 2014, we recruited 105 eligible patients, 60 (57%) of whom were male and 45 (43%) female; their ages ranged from 18 to 81 years (mean age 27·9). In all, 54 subjects (51%) were treated as inpatients and 51 (49%) as outpatients. Within the inpatient group, 30 received ceftriaxone and azithromycin (dual therapy), and 24 were given ceftriaxone alone (monotherapy). In the outpatient group 24 were treated with azithromycin and cefixime (dual therapy) and 27 with azithromycin alone (monotherapy) (**Fig 1**).

Seventeen patients were excluded from FCT analysis for the following reasons: eight had been treated with antibiotics prior to enrollment; nine failed to properly measure their vital signs; and six were switched to treatment regimens other than the antibiotics initially assigned (some cases fell under more than one exclusion criterion). Blood cultures obtained at enrollment revealed an expected antibiotic susceptibility profile for the pathogens, with a high proportion of samples being resistant or partially sensitive to NA (93%) and ciprofloxacin (89%). Resistance to ceftriaxone was observed in one case (1%). Six patients refused to undergo a repeated blood culture on day three and were excluded only from bacteremia duration analysis. Patients in each group did not differ in their clinical presentation in terms of age and gender distribution, symptoms and signs or blood tests, with the exception of diarrhea, which was more common in the group receiving a combination of azithromycin and ceftriaxone. Similarly, patients did not differ in terms of the distribution of the causative agent (*S*. Typhi versus *S*. Paratyphi) and in the degree of its resistance to ciprofloxacin between the groups (**Table 1**).

**Table 1:** Patient characteristics upon enrollment.

	Azithromycin (n = 27)		Ceftrixaone (n = 24)		Azithromycin + ceftriaxone (n = 30)		Azithromycin + cefixime (n = 24)		p-value	Significance
**Demographics**	** **	** **	** **	** **	** **	** **	** **	** **	** **	** **
**Age (years)**	**Mean**	**± STD**	**Mean**	**± STD**	**Mean**	**± STD**	**Mean**	**± STD**		
	29·44	15·67	25·50	7·84	28·63	11·39	27·67	9·37	0·652	ns
**Gender (female)**	**n**	**Percent**	**n**	**Percent**	**n**	**Percent**	**n**	**Percent**		
	9	33%	14	58%	13	43%	9	37%	0·304	ns
**Signs and symptoms**										
	**n**	**Percent**	**n**	**Percent**	**n**	**Percent**	**n**	**Percent**		
**Chills**	16	59%	20	83%	24	80%	18	75%	0·091	ns
**Headache**	26	96%	19	79%	21	70%	22	91·67%	0·094	ns
**Cough**	1	4%	5	21%	3	10%	5	21%	0·307	ns
**Sweating**	8	30%	13	54%	10	33%	12	50%	0·516	ns
**Myalgias**	5	19%	3	13%	4	13%	8	33%	0·329	ns
**Malaise**	16	59%	11	46%	18	60%	12	50%	0·182	ns
**Arthralgia**	4	15%	2	8%	3	10%	4	17%	0·810	ns
**Anorexia**	10	37%	12	50%	14	47%	12	50%	0·753	ns
**Nausea**	11	41%	13	54%	17	57%	10	42%	0·233	ns
**Vomiting**	1	4%	4	17%	5	17%	2	8%	0·290	ns
**Abdominal pain**	4	15%	2	8%	4	13%	4	17%	0·805	ns
**Diarrhea**	4	15%	6	25%	12	40%	1	4%	0·003	**
**Constipation**	3	11%	3	13%	5	17%	1	4%	0·391	ns
**Rash**	0	0%	0	0%	0	0%	2	8%	0·106	ns
**Hepatosplenomegaly**	5	19%	10	42%	8	27%	5	21%	0·325	ns
**Abdominal tenderness**	3	11%	0	0%	3	10%	1	4%	0·248	ns
**Lab tests**										
	**Mean**	**± STD**	**Mean**	**± STD**	**Mean**	**± STD**	**Mean**	**± STD**		
**Hemoglobin**	13·94	1·82	13·52	1·39	14·37	1·47	14·27	1·90	0·264	ns
**WBC**	8737·50	2959·56	7115·83	2789·81	8302·76	3612·13	7263·64	2276·08	0·178	ns
**Neutrophils (% of WBC)**	61·71	8·60	63·42	11·33	68·72	11·26	63·55	12·00	0·103	ns
**Lymphocyte (% of WBC)**	32·79	7·38	32·13	9·92	28·10	10·02	31·45	10·19	0·272	ns
**CRP**	37·10	31·19	51·92	43·24	45·32	34·35	34·89	31·17	0·379	ns
**ESR**	14·83	11·55	18·67	12·95	21·00	10·83	16·73	10·09	0·568	ns
**Microbiology**										
	**n**	**Percent**	**n**	**Percent**	**n**	**Percent**	**n**	**Percent**		
***S*. Typhi**	14	52%	15	63%	18	60%	11	46%	0·621	ns
***S*. Paratyphi**	13	48%	9	38%	12	40%	13	54%	0·621	ns
**Ciprofloxacin sensitivity**	1	4%	3	13%	2	7%	2	8%	0·442	ns

**Fig 2:**
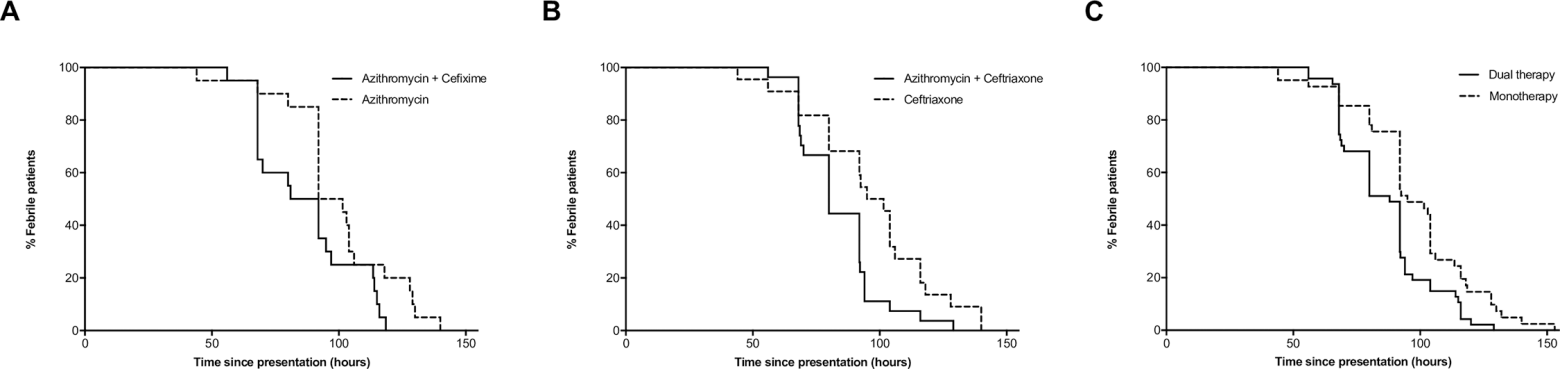
**Kaplan-Meir curve of fever clearance time (FCT) in dual antibiotic therapy versus monotherapy**. A. Time to defervescence for the outpatient study arms; B. Time to defervescence for the inpatient study arms; C. Time to defervescence for the two study arms receiving monotherapy versus the two study arms receiving dual therapy. Dashed lines represent monotherapy, continuous lines represents dual therapy.

FCT was significantly shorter for the 47 patients who received combination therapy compared to the 41 patients who received a single-agent regimen (median values of 88 versus 95 hours, respectively, p = 0·004) (**Fig 2**). Bacteremia was detected in blood cultures drawn on day three for 8/47 (17%) patients who received monotherapy, seven of whom were treated as outpatients and one as inpatient, versus 2/51 (4%) who received combination therapy, both as outpatients (p = 0·045). All patients who were still bacteremic on day three had a negative blood culture on day five. Similarly, when outpatients were analyzed separately, median FCT was 96·75 hours for the oral azithromycin arm versus 86·5 for the combination of azithromycin and cefixime (p = 0·042). For inpatients, median FCT was 98·25 hours for the intravenous ceftriaxone arm versus 80 hours for the combination of ceftriaxone and azithromycin (p = 0·014). It is noteworthy that FCT did not differ significantly between patients infected with *S*. Typhi and *S*. Paratyphi (p = 0·118 for the four study arms pooled together; p = 0·424, 0·212, 0·600, 0·155 for the azithromycin, ceftriaxone, azithromycin+ceftriaxone and azithromycin+cefixime arms, respectively).

Fifty-five participants, who had their phone numbers listed on the medical records, were contacted by the hospital’s Community Health Department after completion of the antibiotic regimen to check for relapse of the disease. They were requested to return to the OPD for a follow-up evaluation a month after convalescence and give a stool sample to assess fecal carriage of the pathogen. Only 19 patients complied with our request to provide stool samples, three from the azithromycin arm, five from the ceftriaxone arm, five from the combined azithromycin and ceftriaxone arm and six from the combined azithromycin and cefotaxime arm, none of them were found to be carriers.

No fatalities, late relapses or drug-related adverse effects were recorded.

## Discussion

Typhoid Fever is a salient cause of morbidity and mortality in the Indian subcontinent, and reflects many aspects of health economics. Being the most common etiology for bacteremia in this part of the world, it places a substantial burden on hospitals and outpatient clinics on the operational level. On the strategic level, by spreading from person to person through contaminated food and water sources it betrays the shortcomings of inadequate infrastructures and poor sanitation, and the precarious use of antibiotics. Treating TF in the Indian subcontinent has become challenging due to rising multidrug resistance. The appropriate choice of an antibiotic regimen for the treatment of TF is not only crucial for an efficient and timely elimination of the disease, but is also for containing its dispersion through control of both the chronic carriage of the pathogen and the emergence of resistance to antibacterial agents.

We decided to combine antimicrobial agents as a strategy for enhancing therapeutic efficacy and reducing the emergence of drug resistance and its rate of transmission. For this purpose, we chose two commonly prescribed antibiotics, azithromycin and third-generation cephalosporins, which are widely used together in infections such as pneumonia and sexually transmitted diseases. However, as opposed to the latter infections, where the rationale for co-administration lies in extending the spectrum of antibiotic coverage for an infection caused by an unknown agent, here the pathogen is known, but its kinetics during the course of infection confer an added value to the combination of antibiotics. Initially, a major part of the bacteria occupies the extracellular compartment, i.e. the blood; hence blood culture positivity rate is the highest at this stage, reaching approximately 80%. Later in the course of infection, the bacteria shift to the intracellular compartment, so that the probability of isolating the pathogen in blood cultures diminishes considerably. We hypothesized that the co-administration of cephalosporins and azithromycin would confer synergism due to their pharmacokinetic attributes, which suggest complimentary modes of action. While cephalosporins remain in the extracellular compartment and thereby effectively eliminate bacteremia, azithromycin readily penetrates the intracellular compartment to eradicate the pathogen in the reticuloendothelial niche [19,20]. The high efficacy of fluoroquinolones before the development of NA-resistant strains can perhaps be attributed to their excellent distribution in both the intra- and extracellular compartments.

Each of these antibiotics is also used separately to treat TF, albeit with known limitations. Attempts to compare their efficacy in the treatment of TF in randomized controlled studies yielded conflicting results, which were susceptible to temporal and geographical variations. A preponderance of the evidence suggests that both ceftriaxone and azithromycin are comparable to previous antibiotic agents when the duration of treatment is adequate [10,11,13,21–24]. Nevertheless, azithromycin did show a slight advantage over ceftriaxone in terms of reduced relapse rates [10]^,^[25]. In contrast, cefixime, although effective in some studies [26–31], was found to be inferior to commonly administered antibiotic agents in other studies and was hence not recommended for use as a single agent for the treatment of TF [32]^,^[33]. Notably, current guidelines for *in vitro* susceptibility testing involving disc diffusion and blood concentrations do not accurately reflect the clinical response to azithromycin [22], thus calling for the revision of breakpoint recommendations and reassessment of previously collected data [10].

Our study suggests that the combination of third-generation cephalosporins and azithromycin may confer a more effective therapy, reducing the time to defervescence and to the clearance of bacteremia. We have shown that dual antibiotic therapy supersedes monotherapy in terms of FCT by approximately 12 hours, both in in- and outpatient settings. Our mean time to defervescence coincided with previous studies conducted in similar TF-endemic populations [22–24,32,34–36].

Our trial shows some significant strengths: it is a prospective trial, carried out over a period of two years and meeting rigorous inclusion criteria. Only blood culture-confirmed cases were eligible to avoid biases that could arise from the inclusion of patients presenting with symptoms similar to those of TF, and for which an alternative diagnosis was eventually found [18,37]. All the same, some of our study’s limitations ought to be addressed: patients were allocated to study arms by alternation, which does not qualify as a purely random process. Additionally, our study population consisted of patients 18 years or older, so that its conclusions cannot be applied to a pediatric population. Vital signs were recorded twice daily in concordance with the hospital’s policy, which might have led to overestimation of the actual FCT, as more frequent measurements of body temperature could potentially have resulted in a more accurate approximation. Furthermore, a combined therapy of two drugs with different action mechanisms may potentially affect relapse and the chronic carriage of pathogens, which is of great importance in TF endemic regions. Unfortunately, our sample size was too small to detect such effects. As often happens in rural areas in developing countries, due to logistic and communication problems and a lack of incentives, many patients did not return for follow-up visits after recovery. Therefore, data regarding relapse rates and chronic carriage of the disease were partial.

It should also be noted that the Israeli study performed on travelers returning from Nepal demonstrated a greater gap in FCT between patients receiving dual antibiotic therapy and those receiving a single antibiotic agent [17]. This could be attributed to a number of factors, all host-related, such as the difference between an indigenous population constantly exposed to varying loads of the pathogen in their environment and a naïve population [38]; or disease-related factors, such as antibiotic resistance profiles and the diversity of causative bacteria in the former case as compared to an infection by one strain of *S*. paratyphi A in the latter. Finally, the study conducted on travelers was not randomized controlled, which can lead to biased results.

From a clinical point of view, we propose a new paradigm to the treatment of TF, which shortens the duration of the disease and can potentially prevent the emergence of resistance to antibiotics following a synergistic mode of action. Since many afflicted patients in endemic areas are poor, and thus cannot afford hospitalization and healthcare costs, reducing the length of therapy is critical. Furthermore, the added value of dual antibiotic therapy over monotherapy is evident in the outpatient setting as well, and may thus be even more relevant to deprived communities, where healthcare services and intravenous treatment are scarce. Our findings warrant further research to determine whether the results can be reproduced and applied to other indigenous populations. They would also help assess whether a combination of two antibiotic agents that act on both the extra- and the intracellular compartments can decrease treatment failure, relapse and carriage rates, and reduce the emergence of resistant bacterial strains [39]^,^[40]. Such beneficial effects would prompt a shift in the current approach to TF treatment and translate into better and more efficient treatment.

## Supporting information

S1 Checklist CONSORT checklist for the trial. (DOC)

S1 Data Study database. (XLSX)

S1 Protocol Study protocol. (PDF)
