# Supplementary material for: Open label comparative trial of mono versus dual antibiotic therapy for Typhoid Fever in adults
Source: PLoS Negl Trop Dis. 2018 Apr 23;12(4):e0006380. doi: 10.1371/journal.pntd.0006380 (PMC5912710; doi:10.1371/journal.pntd.0006380)
Supplement: S1 Protocol — (PDF) [file pntd.0006380.s003.pdf]

Typhoid Fever: Combined vs. Single Antibiotic Therapy

Niv Zmora,Sudeep Shrestha,Ami Neuberger,Yael Paran,Rajendra Tamrakar,Ashish Shrestha,Surendra K. Madhup,TRS Bedi,Rajendra Koju,Eli Schwartz

Abstract

Typhoid Fever: Combined vs. Single Antibiotic Therapy

Citation: Niv Zmora,Sudeep Shrestha,Ami Neuberger,Yael Paran,Rajendra Tamrakar,Ashish Shrestha,Surendra K. Madhup,TRS Bedi,Rajendra Koju,Eli SchwartzTyphoid Fever: Combined vs. Single Antibiotic Therapy. **protocols.io**
dx.doi.org/10.17504/protocols.io.kdccs2w
Published: 19 Oct 2017

Materials

500 mg Azithromycin Dihydrate
orb322360

1 g Ceftriaxone Sodium
orb320831

Protocol

Purpose

1. The current study goal is to examine the effect of Cephalosporins, Azithromycin and the combination of both on typhoid fever therapy in endemic population.
The investigator's hypothesize that the combination of azithromycin and ceftriaxone may prove superior to each drug, ceftriaxone or azithromycin, alone.

Description

2. Typhoid Fever (TF) is a highly prevalent infection in the Indian subcontinent. Due to multidrug resistant strains in these areas, third generation cephalosporins, such as ceftriaxone, are the treatment of choice. However, the latter regimen exhibits a slow response with mean time of 5 to 7 days or even longer to defervescence, which could be attributed to poor penetration capability of the drug into cells, and thus difficulty to eradicate the bacteria from the intracellular niche.
Attempts have been made to overcome this setback by introducing alternative antibiotic regimens, such as azithromycin. However studies comparing between azithromycin and a third-generation cephalosporin for the treatment of typhoid fever in adult population in the Indian subcontinent are lacking.
Over the last few years our approach towards non-immunized travelers, who acquired typhoid fever in the Indian subcontinent, was to administer a combination therapy of intravenous ceftriaxone with oral azithromycin. The rationale of this dual regimen was its pharmacokinetic profile, which suggests a complimentary action of the two agents - ceftriaxone on the extracellular compartment and azithromycin on the intracellular compartment. Moreover, in our clinical experience, preliminary published data has proven combination therapy significantly superior to ceftriaxone alone albeit in a small group of travelers.
In the current study the investigators intend to compare the efficacy of ceftriaxone vs. azithromycin and vs. combined therapy of both agents for the treatment of uncomplicated typhoid fever in terms of time to defervescence.
4 different treatment strategies will be examined (as mentioned in the arm section). All participants will be checked for vital signs, will undergo physical examination, ECG, laboratory testing, blood, urine and stool culture and tests for susceptibility to antibiotics.

This is an open access protocol distributed under the terms of the Creative Commons Attribution License, which permits unrestricted use, distribution, and reproduction in any medium, provided the original author and source are credited

Study design and participants

3. A multi-arm, parallel, open-label, randomized controlled trial will be conducted on adult patients, 18 years of age or older, who will attended Dhulikhel Hospital, Nepal, between October 2012 and October 2014.
Subjects with blood cultures positive for S. Typhi or S. Paratyphi will be eligible. Exclusion criteria will include known allergies to cephalosporins or macrolides, inability to swallow oral medications, antibiotic treatment received up to four days prior to admission, significant underlying illness and pregnancy or lactation at the time of enrollment.

Randomization process and treatment arms

4. Febrile adult patients attending the emergency room (ER) or outpatient department (OPD), who will be clinically suspected of having TF by Dhulikhel Hospital's physicians and who will comply with the study inclusion and exclusion criteria will be given a detailed explanation regarding the study, and will provide written informed consent. Venous blood cultures will be drawn prior to antibiotic treatment at the time of enrollment.

Assigned Interventions

5. Patients will be initially assigned into either an inpatient or an outpatient setting, according to the severity of their symptoms and their personal preference. They will then be allocated to two treatment arms, monotherapy versus dual therapy, according to the order of arrival in an equal allocation ratio. Treatment arms for inpatients will be an intravenous 2-gram dose of ceftriaxone once daily (OD) versus a combination of an intravenous 2-gram dose of ceftriaxone OD plus oral azithromycin 500 mg OD; treatment arms for outpatients will be oral azithromycin 500 mg OD versus a combination of oral azithromycin 500 mg OD plus oral cefixime 400 mg OD. Neither the patients nor the medical personnel will be blinded to the assignment into groups.

Data collection

6. Once blood culture results will be available, patients with proven S. Typhi or S. Paratyphi bacteremia will be included in the study and will be asked to fill out a demographic questionnaire. Patients with negative cultures will be excluded from the study and receive standard care.
Demographic data, including age, gender, occupation and place of residence, along with presenting symptoms and medical history will be collected through a questionnaire. Physical examination will be performed by a qualified physician.
Inpatients will have their vital signs taken and undergo physical examination twice daily by the Dhulikhel Hospital Internal Medicine ward staff. The vital signs of outpatients will be recorded at 12-hour intervals by trained community medical auxiliaries during house calls, or the patients can alternatively attend nearby clinics and pharmacies.
Blood tests and cultures will be collected initially at enrollment and then on day three. Patients who will have persistent bacteremia on day three will have a third blood culture drawn on day five as well. Blood will be cultured in the hospital's microbiological laboratory by use of BACTEC radiometric blood cultures. Testing of isolates for susceptibility to various antibiotics will be carried out by the disc diffusion method, and in case of resistance to the assigned regimen, treatment will be switched accordingly and patient will be excluded from the study.
One month following discharge patients will be asked to return for vital signs recording, physical examination and stool cultures in order to check for cases of relapse and assess fecal carriage of the pathogen.

Outcome Measures

This is an open access protocol distributed under the terms of the Creative Commons Attribution License, which permits unrestricted use, distribution, and reproduction in any medium, provided the original author and source are credited

7. Antibiotic treatment will be administered for 7 days or 72 hours following defervescence (whichever was longer). Inpatients will be discharged from hospitalization 24-48 hours following defervescence or upon their request, and will be asked to complete a 7-day antibiotic course. When discharged, the patients will be given oral cefixime for the remainder of the treatment instead of the intravenous ceftriaxone. In case of persistent fever, treatment will be extended as deemed necessary by the attending physician. The primary endpoint of our trial will be fever clearance time (FCT), defined as the time required to achieve oral temperature of 37.5 degrees Celsius or below after the beginning of antibiotic treatment. The use of paracetamol will be restricted to pain relief and not fever alleviation, and FCT was determined after confirmation that the patient had not taken paracetamol 12 hours prior to vital signs measurement. Secondary endpoints will be treatment failure, defined as the need to switch antibiotic treatment according to physician's decision, bacteremia clearance time, development of TF-related complications, late relapse, fecal carriage and adverse drug reactions.

This is an open access protocol distributed under the terms of the Creative Commons Attribution License, which permits unrestricted use, distribution, and reproduction in any medium, provided the original author and source are credited
